# Acute evacuation of 54 intracerebral hematomas (aICH) during the microsurgical clipping of a ruptured middle cerebral artery bifurcation aneurysm—illustration of the individual clinical courses and outcomes with a serial brain CT/MRI panel until 12 months

**DOI:** 10.1007/s00701-024-05902-9

**Published:** 2024-01-17

**Authors:** Anniina H. Autio, Juho Paavola, Joona Tervonen, Maarit Lång, Antti-Pekka Elomaa, Terhi J. Huuskonen, Jukka Huttunen, Virve Kärkkäinen, Mikael von Und Zu Fraunberg, Antti E. Lindgren, Timo Koivisto, Jouni Kurola, Juha E. Jääskeläinen, Olli-Pekka Kämäräinen

**Affiliations:** 1 Neurosurgery, NeuroCenter, Kuopio University Hospital, PL 100, 70029 Kuopio, Finland; 2 Institute of Clinical Medicine, School of Medicine, Faculty of Health Sciences, University of Eastern Finland, Kuopio, Finland; 3 Neurointensive Care Unit, Kuopio University Hospital, Kuopio, Finland; 4 Department of Neurosurgery, Oulu University Hospital, Oulu, Finland; 5 Research Unit of Clinical Medicine, University of Oulu, Oulu, Finland; 6 Clinical Radiology, Kuopio University Hospital, Kuopio, Finland; 7 Center for Prehospital Emergency Care, Kuopio University Hospital, Kuopio, Finland

**Keywords:** Aneurysmal intracerebral hematoma, Brain tissue outcome, EMS Emergency medical services, Individual serial brain imaging panel, Microsurgical evacuation and clipping, Perihematomal edema

## Abstract

**Purpose:**

In aneurysmal intracerebral hemorrhage (aICH), our review showed the lack of the patient’s individual (i) timeline panels and (ii) serial brain CT/MRI slice panels through the aICH evacuation and neurointensive care until the final brain tissue outcome.

**Methods:**

Our retrospective cohort consists of 54 consecutive aICH patients from a defined population who acutely underwent the clipping of a middle cerebral artery bifurcation saccular aneurysm (Mbif sIA) with the aICH evacuation at Kuopio University Hospital (KUH) from 2010 to 2019. We constructed the patient’s individual timeline panels since the emergency call and serial brain CT/MRI slice panels through the aICH evacuation and neurointensive care until the final brain tissue outcome. The patients were indicated by numbers (1.–54.) in the pseudonymized panels, tables, results, and discussion.

**Results:**

The aICH volumes on KUH admission (median 46 cm^3^) plotted against the time from the emergency call to the evacuation (median 8 hours) associated significantly with the rebleeds (*n*=25) and the deaths (*n*=12). The serial CT/MRI slice panels illustrated the aICHs, intraventricular hemorrhages (aIVHs), residuals after the aICH evacuations, perihematomal edema (PHE), delayed cerebral injury (DCI), and in the 42 survivors, the clinical outcome (mRS) and the brain tissue outcome.

**Conclusions:**

Regarding aICH evacuations, serial brain CT/MRI panels present more information than words, figures, and graphs. Re-bleeds associated with larger aICH volumes and worse outcomes. Swift logistics until the sIA occlusion with aICH evacuation is required, also in duty hours and weekends. Intraoperative CT is needed to illustrate the degree of aICH evacuation. PHE may evoke uncontrollable intracranial pressure (ICP) in spite of the acute aICH volume reduction.

## Introduction

Aneurysmal subarachnoid hemorrhage (aSAH) is a complex and potentially critical systemic condition, requiring Emergency Medical Service (EMS) care, immediate CT diagnosis and transfer to the multidisciplinary neurointensive care [32, 75]. Aneurysmal intracerebral hemorrhage (aICH) is a frequent and damaging complication [2, 3, 13, 25, 47, 79, 84], a potential cause of sudden death [43] or brain death during neurointensive care [40]. Human brain tissue is soft with low tensile strength [31]. Arterial blood jet from the ruptured aneurysm wall creates a brain cavity filled with an aICH clot, a permanent transsecting local injury in the brain tissue connectome, a cerebrospinal fluid (CSF) cavity in later neuroimaging [5]. Re-bleeding [2, 56, 68, 71] before the aneurysm occlusion may increase (i) the risk of aICH or (ii) the volume of an existing aICH.

Neurovascular teams serving their populations often face the decision which aICHs, regarding, site and volume in which aSAH patients, regarding acute condition [60], age and previous condition [105] in which time since the ictus and EMS call should be microsurgically evacuated. Ultra-early evacuation of a large aICH with imminent brain herniation [60] may be lifesaving [80]. The aICH clot evacuation is palliative only, aimed to reduce increased intracranial pressure (ICP), compressive and tensile brain tissue stress, neuroinflammation [8, 70, 81], perihematomal edema (PHE) with brain injury [9, 46, 59], delayed cerebral ischemia (DCI) [41, 62], and time to rehabilitation [10]. Many secondary factors, including complications of the aICH evacution with saccular intracranial aneurysm (sIA) clipping, may aggravate the final brain tissue injury around the aICH cavity [2].

It seems that acute aICHs are sidelined in clinical neuroacutology and literature in many ways. It would be straightforward to relieve the brain from the ‘poisonous clot’ soon after the EMS call [55, 58, 63, 70]. Otherwise, lysing erythrocytes start to release hemoglobin, a strong culprit in secondary brain damage; haptoglobin therapy against cell-free hemoglobin is now considered [21]. However, a new paradigm has evolved: acute endovascular treatment (EVT) of aneurysm followed by a delayed aICH evacuation [50]. A hybrid operating room [23, 27] might decrease the delay, with intraoperative CT showing promptly the true degree of aICH removal. Many articles portray aICHs and their evacuations, and acute, delayed and longterm brain tissue injuries with words, phrases, numbers, scales and scores, etc. The frequently expressed modified Fisher scale does not specify aICHs (e.g., location, size, shape, extensions) at all [49]. PHE with secondary brain injury around aICH receives virtually no attention as compared to ‘vasospasm’ and DCI. It is difficult to find aICH articles with *all patients’* individual timeline panels and serial brain CT/MRI slice panels from the ictus until one to three years (final brain tissue outcome) [2].

Our study cohort consists of 54 consecutive aICH patients from a defined population who acutely underwent a microsurgical clipping of the ruptured middle cerebral artery bifurcation (Mbif) sIA together with the aICH evacuation. The Mbif site was selected for a homogeneous study group with one hemisphere affected only. We constructed a serial brain CT/MRI panel for the 54 patients [2]: two axial CT slices representative of the aICH volume, CT 1. before and CT 2. after the evacuation. The latest available CT slice or MRI slice was selected for the 42 survivors at about 12 months.

Our aims were to illustrate in real life to the clinician readers (evaluate yourself)

- the shapes and volumes of the 54 Mbif aICHs,
- the impact of verified or clinically suspected re-bleeds,
- the extent of the aICH evacuations in terms of residual hematomas,
- the development of brain edema, PHE, and DCI, and
- the extent of brain tissue injuries around the aICH cavity at about 12 months (*final brain tissue outcome)* in the survivors.

## Methods and materials

### Kuopio University Hospital (KUH) and Emergency Medical Services (EMS) in Eastern Finland

KUH, one of the five university hospitals in Finland, is an academic, non-profit, publicly funded tertiary center, which serves a defined population (805,133 in 2019) in Eastern Finland. The overall KUH catchment area, during the study period 2010–2019, contained four Central Hospitals with the districts of their own, each with 24/7 neuroacutology, CT services, intensive care, and neurorehabilitation. The road transfer distances between KUH and each Central Hospital range from 141 to 162 km [2]. During the study period the KUH catchment area was served by 74 (1 per 10 900 citizens) advanced level and basic level EMS units and a physician-manned helicopter EMS Unit (HEMS). Dispatching and mission control is Global Positioning System (GPS) based [57].

### KUH NeuroCenter and subarachnoid hemorrhage (SAH) in Eastern Finland

At KUH Neurosurgery, at least two neurosurgeons were on duty at all times, with on-line phone and teleconsultation of digital imaging from the referring hospitals. In principle, all patients with SAH would be acutely transferred to KUH for neurointensive care, 4-vessel catheter angiography and/or CT angiography, and neurosurgical and endovascular interventions, including Hunt & Hess (HH) 4–5 patients [2, 3, 40]. Depending on the patient’s condition and CT findings, intubation (if not performed) and a physician, anesthesiologist or intensivist attending the patient during the transfer were agreed.

At KUH, a dedicated team of neurointensivists, neurosurgeons, and interventional neuroradiologists coordinated the aSAH treatment. The KUH Neurovascular Group provided microsurgical or endovascular occlusion of the ruptured aneurysm and evacuated significant intracerebral hematomas (aICHs) with immediate microsurgery [2, 3]. Temporal arterial occlusion, indocyanine green angioprahy (ICG) and micro-Doppler were used. Intraoperative CT [30, 74] to verify the degree of aICH clot removal was not available.

The KUH aSAH neurointensive care protocol in 2010–2019 followed international recommendations in detail, aimed to prevent further brain damage due to re-bleeding, increased intracranial pressure (ICP below 20 mmHg; cerebral perfusion pressure at 60–70 mmHg), hydrocephalus, electrolyte disturbances, seizures, cardiac and pulmonary dysfunction, fever, hyperglycemia, and development of delayed brain ischemia. Tranexamic acid (1g × 4 i.v.) was routinely started after the SAH-verifying CT and continued until the occlusion of the ruptured sIA during the study period 2010–2019. The protocol included: external ventricular drainage (EVD), ventricular or parenchymal ICP monitoring, endovascular procedures and intra-arterial nimodipine infusion in delayed brain ischemia, and decompressive craniectomy (DC). KUH neurointensive care monitoring data is stored in the prospective Finnish Intensive Care Consortium database [61].

### Kuopio Intracranial Aneurysm Patient and Family Database

The database, prospective since 1995, contains all cases of unruptured and ruptured intracranial aneurysms (IAs) admitted to KUH since 1980. A dedicated, full-time nurse administrates the database, interviews all new IA patients, including their family history, and arranges the follow-ups. The clinical data, including prescribed medicines, hospital diagnosis, and causes of death have been fused from the national registries, using the Finnish personal codes. Three 1. degree relatives with a diagnosed sIA disease form an sIA family. We have characterized our aSAH patients from many clinical points of view and for long-term outcome [2, 3, 40, 72].

### Final study population

**Fig. 1:**
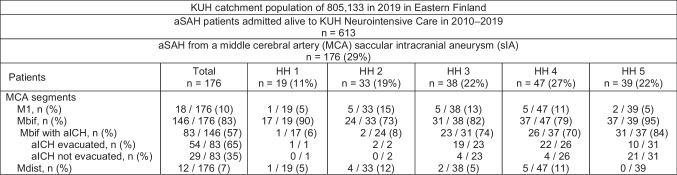
Flowchart. A total of 613 consecutive patients were acutely admitted—within 24 h from the CT diagnosis of the first subarachnoid hemorrhage (SAH) – to the neurointensive care at the Kuopio University Hospital (KUH) between 2010 and 2019 from a defined population. A total of 146 patients had a saccular intracranial aneurysm (sIA) on the middle cerebral artery bifurcation (Mbif), with 83 / 146 (57%) causing an intracerebral hematoma (aICH). The 176 clinical date point timelines were re-constructed until the death (*n*= 56 / 176) or the last follow-up. The final study population consists of the 54 patients with the evacuation of the aICH during the microsurgical clipping of the Mbif sIA. Abbreviations: aICH, aneurysmal intracerebral hematoma; aSAH, aneurysmal subarachnoid hemorrhage; CT, computed tomography; HH, Hunt and Hess scale; KUH, Kuopio University Hospital; M1, middle cerebral artery M1 trunk segment; Mbif, middle cerebral artery bifurcation; Mdist, middle cerebral artery peripheral segments; SAH, subarachnoid hemorrhage; sIA, saccular intracranial aneurysm

**Fig. 2:**
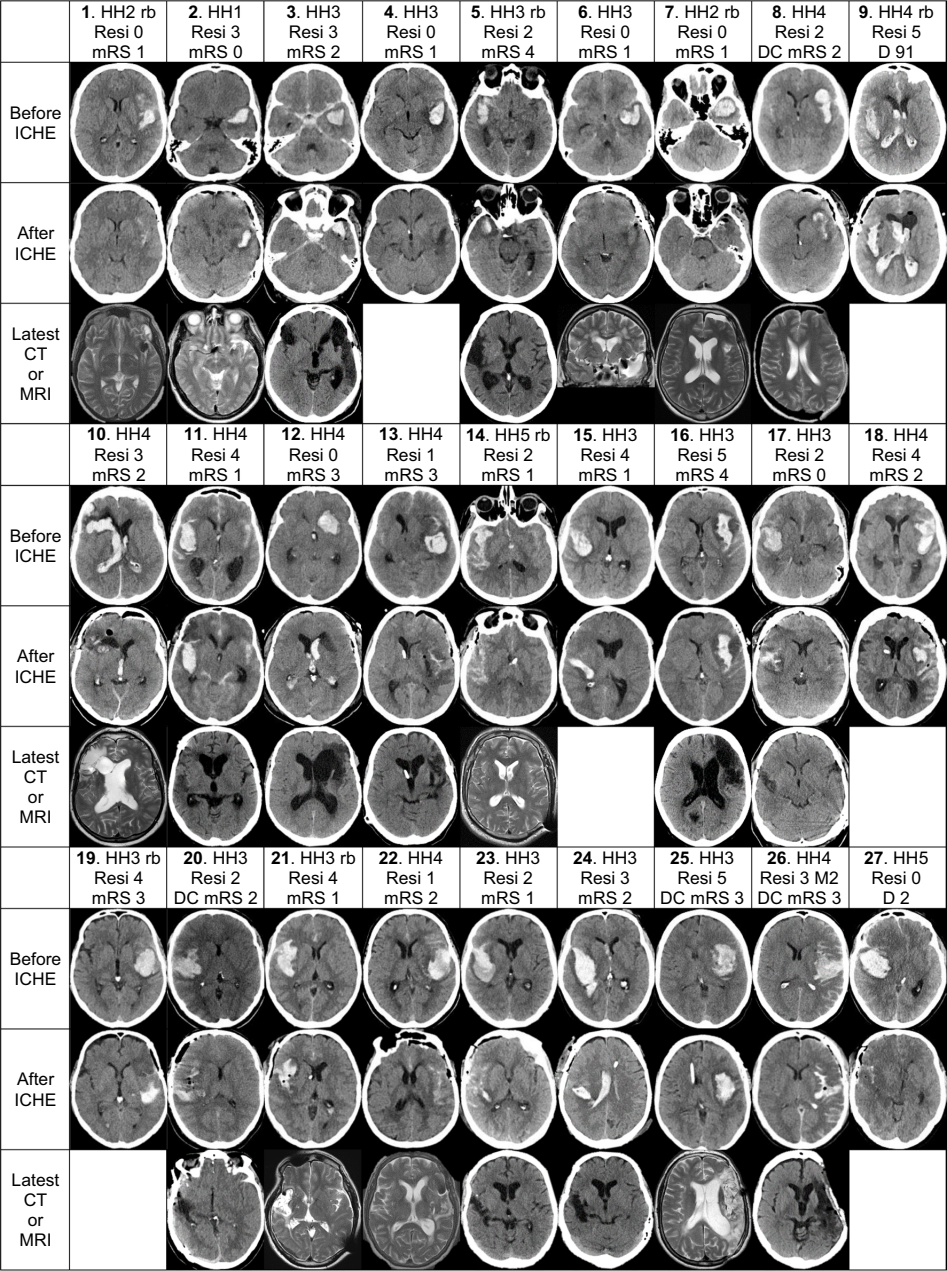

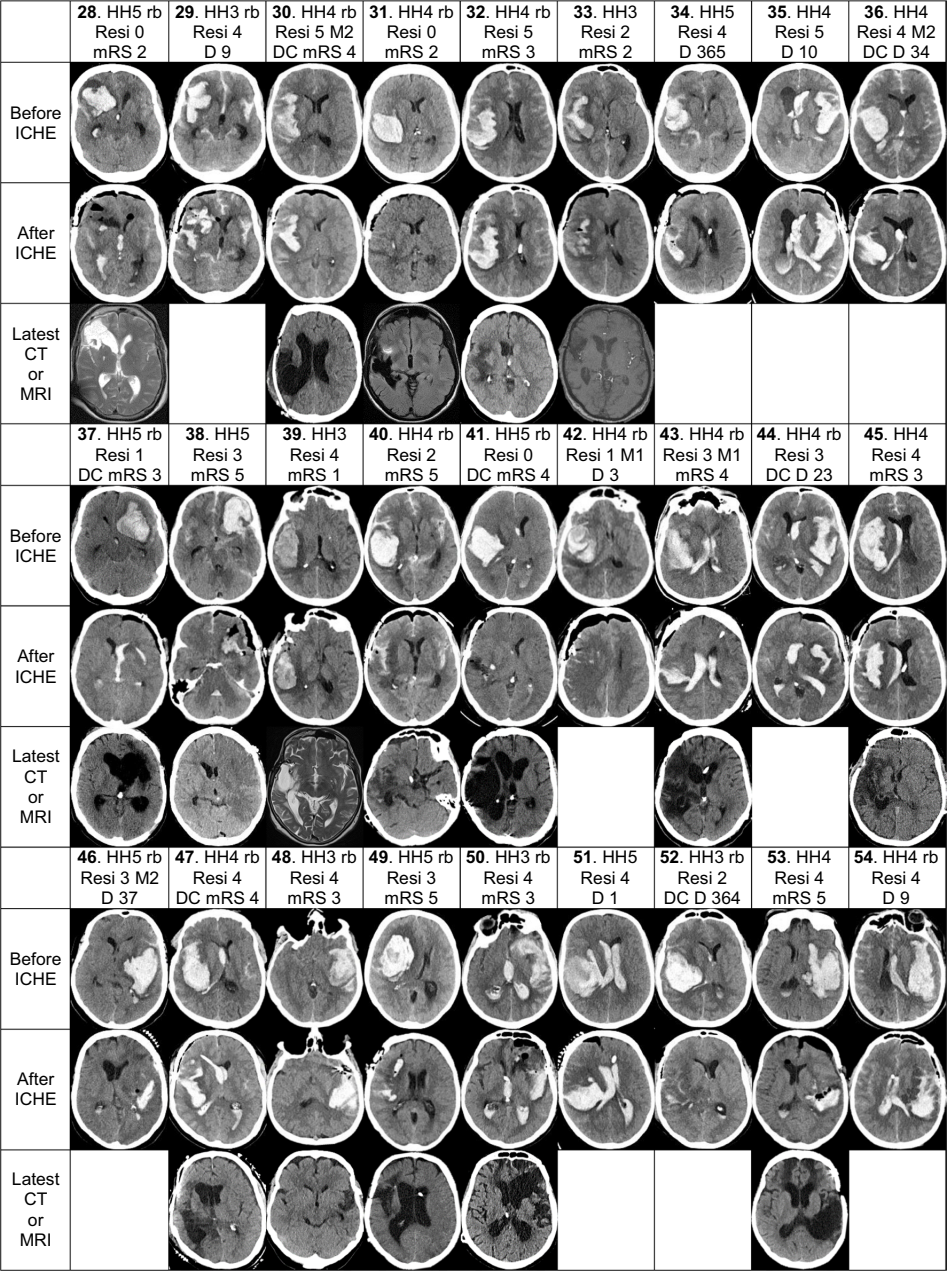
Individual CT/MRI panels of the 54 patients. The serial CT/MRI scan panels of the 54 patients with an acute intracerebral hematoma (aICH) from a saccular intracranial aneurysm (sIA) on the middle cerebral artery (MCA) bifurcation (Mbif) (Flowchart in Fig 1). After the 112 call, the 54 aSAH patients were transferred by the emergency medical services (EMS) to the neurointensive care in the Kuopio University Hospital (KUH). A total of 25 (46%) patients had a clinical or CT-verified re-bleed (rb) before the sIA occlusion. All 54 patients (Patients 1.–54.) underwent the microsurgical clipping of the ruptured Mbif sIA with the intention to remove the aICH. Of the 54 patients, 17 (32%) had also an intraventricular hematoma (aIVH). For each patient, two axial CT slices were selected: CT 1. before the clipping and CT 2. after the clipping. The 54 CT 1. slices are arranged according to the increasing largest area of the aICH on admission. The latest available slice (CT or MRI) was selected for the survivors at about 12 months (not available in the Patients 4. 15. 18. 19.). The white data box contains: the patient number (1.–54.); the Hunt and Hess scale (HH) on admission; rb, re-bleed before sIA occlusion; decompressive craniectomy (DC; *n*=11); mRS 0 to 5 at about 12 months for the 42 survivors; D and days to death for the 12 deceased patients. The white box also contains the visual estimate of the aICH volume reduction (Resi) as sizes of the residuals (0–5) graded as follows: 0—no residual (*n*=9); 1—patchy small residual (*n*=4); 2—patchy residual (*n*=9); 3—small solid residual (*n*=10); 4—partial solid residual (*n*=16); 5—all solid left (*n*=6). M1 denotes the M1 occlusion in two patients (42. 43.) and M2 denotes the M2 branch occlusion in four patients (26. 30. 36. 46.) in the postoperative angiography. Abbreviations: aICH, aneurysmal intracerebral hematoma; aIVH, aneurysmal intraventricular hematoma; CT, computed tomography; D, deceased; DC, decompressive craniectomy; EMS, emergency medical services; HH, Hunt and Hess scale; ICHE, aICH evacuation; KUH, Kuopio University Hospital; M1, middle cerebral artery M1 trunk segment; M2, middle cerebral artery M2 branch segments; Mbif, middle cerebral artery bifurcation; MCA, middle cerebral artery; MRI, magnetic resonance imaging; mRS, modified Rankin Scale; rb, re-bleed before the sIA occlusion; Resi, visual estimate of the aICH volume reduction in the first CT after the evacuation; sIA, saccular intracranial aneurysm

**Fig. 3:**
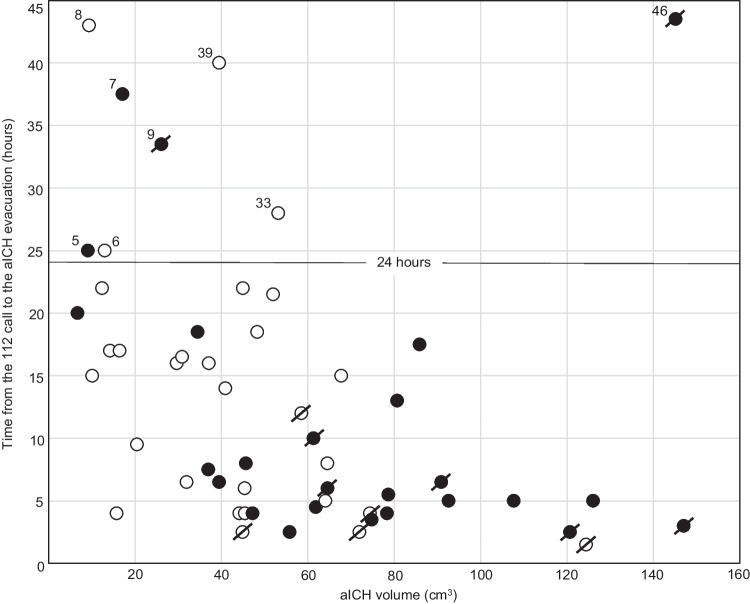
The 54 patients’ aICH volumes in relation to the time elapsed to the aICH evacuation. The 54 patients with their aICH volumes on admission and their individual times from the 112 call to the aICH evacuation during the microsurgical clipping of the ruptured Mbif sIA. The x-axis presents the 54 aICH volumes (cm^3^) and the y-axis presents the corresponding times (hours). The 54 aICH volumes distributed as follows 7–31–46–75–147 cm^3^ and the 54 times 1–4–8–18–43 hours (shortest – 25% quartile – median – 75% quartile – longest). In a total of 8 patients, the times exceeded 24 hours (horizontal line), in association with a small aICH (Patients 5. 6. 7. 8. 9. 33.), good condition (Patient 39.), or poor initial condition (Patient 46.). The time to the sIA clipping significantly shortened with the increasing aICH volume (*p* < 0,001). Re-bleeds (black circle) occurred in the 25 (46%) patients, and 7 (28%) of them deceased ( **/** ). No re-bleeds (white circle) occurred in the 29 (54%) patients, and 5 (17%) of them deceased ( **/** ). The re-bleeds associated significantly with the increasing aICH volume (*p* = 0,006). Abbreviations: aICH, aneurysmal intracerebral hematoma; cm^3^, cubic centimeter; Mbif, middle cerebral artery bifurcation; sIA, saccular intracranial aneurysm

**Fig. 4:**
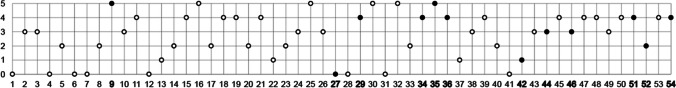
The 54 patients arranged according to their largest aICH area and aICH residual grade. The 54 patients with an acute aneurysmal intracerebral hematoma (aICH) evacuated during the clipping of the ruptured middle cerebral artery (MCA) bifurcation (Mbif) saccular intracranial aneurysm (sIA). The patients (1.–54.) are arranged according to the increasing largest area of the aICH in the preoperative CT scan (Fig 2). The residual aICH volume (Resi) in the first postoperative CT scan was visually graded (0 to 5) as follows: 0 – no residual (*n*=9; 17%); 1 – patchy small residual (*n*=4; 7%); 2 – patchy residual (*n*=9; 17%); 3 – small solid residual (*n*=10; 19%); 4 – partial solid residual (*n*=16; 30%); 5 – all solid left (*n*=6; 11%). The outcome until 12 months is indicated by the dot colour: death (black, *n*=12, patient numbers in bold) and alive (white, *n*=42). Abbreviations: aICH, aneurysmal intracerebral hematoma; CT, computed tomography; Mbif, middle cerebral artery bifurcation; MCA, middle cerebral artery; Resi, visual estimate of the aICH volume reduction in the first CT after the evacuation; sIA, saccular intracranial aneurysm

A total of 176 consecutive aSAH patients with a ruptured sIA on the middle cerebral artery (MCA) were acutely admitted, within 24h from the CT diagnosis of SAH, from the defined Eastern Finnish catchment population to the KUH Neurointensive Care Unit from 2010 to 2019 (Fig. 1). Of the 176 ruptured MCA sIAs, 146 (83%) were on the MCA bifurcation, 83 (57%) of them with an aICH (Fig. 1). The clinical timelines were re-constructed until the death (n= 56 / 176) or the end of 2021 from the data in the Kuopio database and from the national clinical registries [2, 3]. The final study population consisted of the 54 Mbif sIA patients (Patients 1.–54.) with an aICH evacuated during the microsurgical clipping of the ruptured sIA (Table 1, Figs. 1, 2). They had a postoperative CT scan within 5 days after the sIA occlusion. Of the 54 patients, 12 (22%) were deceased at 12 months and 42 (78%) were alive (Table 1, Figs. 2, 3, 4). In addition, we evaluated why the remaining 29 Mbif aICH patients did not undergo an acute aICH evacuation in association with the sIA clipping (Fig. 1).

**Table 1:** The characteristics of the 54 patients with the evacuation of the intracerebral hematoma (aICH) during the microsurgical clipping of the ruptured middle cerebral artery (MCA) bifurcation (Mbif) saccular intracranial aneurysm (sIA). The residual aICH volumes (Resi) in the postoperative CT scans (Fig. 2) were visually graded (0 to 5) as follows: 0 – no residual (*n*=9); 1 – patchy small residual (*n*=4); 2 – patchy residual (*n*=9); 3 – small solid residual (*n*=10); 4 – partial solid residual (n=16); 5 – all solid left (*n*=6)

Variables	Mbif sIA aICH removals n = 54	Resi 0 n = 9 (17%)	Resi 1 n = 4 (7%)	Resi 2 n = 9 (17%)	Resi 3 n = 10 (19%)	Resi 4 n = 16 (30%)	Resi 5 n = 6 (11%)
Females, n (%)	26 (48%)	6 / 9	2 / 4	3 / 9	4 / 10	6 / 16	5 / 6
Median age at aSAH, years	54	50	55	48	50	60	60
Drug treated hypertension, n (%)	19 (35%)	4 / 9	1 / 4	5 / 9	2 / 10	5 / 16	2 / 6
Condition at the EMS contact							
Median GCS	13	9	8	13	14	12	14
Re-bleeds before the sIA occlusion, n (%)	25 (46%)	5 / 9	2 / 4	4 / 9	4 / 10	7 / 16	3 / 6
possibly by symptoms, n	16 / 25	4 / 5	1 / 2	2 / 4	3 / 4	4 / 7	2 / 3
verified by two CTs, n	9 / 25	1 / 5	1 / 2	2 / 4	1 / 4	3 / 7	1 / 3
Median times from the 112 call							
to the KUH arrival, hours	2	2	4	2	3	2	3
to the aICH removal, hours	8	16	6	18	11	5	11
Condition at the KUH arrival							
Hunt & Hess 1–2, n (%)	3 (6%)	2 / 9	0 / 4	0 / 9	1 / 10	0 / 16	0 / 6
Hunt & Hess 3, n (%)	19 (35%)	2 / 9	0 / 4	6 / 9	2 / 10	7 / 16	2 / 6
Hunt & Hess 4, n (%)	22 (41%)	2 / 9	3 / 4	2 / 9	4 / 10	7 / 16	4 / 6
Hunt & Hess 5, n (%)	10 (19%)	3 / 9	1 / 4	1 / 9	3 / 10	2 / 16	0 / 6
aICH							
Median aICH volume, cm^3^	46	17	55	41	55	60	38
The left hemisphere, n (%)	24 (44%)	5 / 9	3 / 4	1 / 9	6 / 10	6 / 16	3 / 6
aIVH, intraventricular clot, n (%)	17 (32%)	3 / 9	1 / 4	1 / 9	4 / 10	6 / 16	2 / 6
The sIA clipping and the aICH removal							
Median sIA size, mm	9,0	11,0	11,5	8,0	8,5	10,0	7,5
Intraoperative re-bleeding, n (%)	16 (30%)	1 / 9	2 / 4	1 / 9	3 / 10	7 / 16	2 / 6
Median temporary MCA occlusion, min	5	5	2	5	5	8	8
Intraoperative Doppler, n (%)	48 (89%)	8 / 9	3 / 4	8 / 9	8 / 10	15 / 16	6 / 6
Intraoperative ICG, n (%)	46 (85%)	7 / 9	4 / 4	7 / 9	9 / 10	13 / 16	6 / 6
Postoperative DSA/CTA, n (%)	51 (94%)	7 / 9	4 / 4	9 / 9	9 / 10	16 / 16	6 / 6
M1 trunk occlusion, n (%)	2 (4%)	0 / 9	1 / 4	0 / 9	1 / 10	0 / 16	0 / 6
M2 branch occlusion, n (%)	4 (7%)	0 / 9	0 / 4	0 / 9	2 / 10	1 / 16	1 / 6
Decompressive craniectomy, n (%)	11 (20%)	1 / 9	1 / 4	3 / 9	2 / 10	2 / 16	2 / 6
Shunt, n (%)	13 (24%)	2 / 9	1 / 4	2 / 9	3 / 10	3 / 16	2 / 6
Median of neurointensive care days	9	8	9	9	8	9	10
Death until 14 days, n (%)	6 (11%)	1 / 9	1 / 4	0 / 9	0 / 10	3 / 16	1 / 6
Alive at 12 months, n (%)	42 (78%)	8 / 9	3 / 4	8 / 9	8 / 10	11 / 16	4 / 6
Median mRS at 12 months	3	2	3	2	4	3	4

*aICH* aneurysmal intracerebral hematoma, *aIVH* aneurysmal intraventricular hematoma, *aSAH* aneurysmal subarachnoid hemorrhage, *CT* computed tomography, *CTA* computed tomography angiography, *DSA* digital subtraction angiography, *EMS* emergency medical services, *GCS* Glasgow Coma Scale, *ICG* indocyanine green angiography, *KUH* Kuopio University Hospital, *M1* middle cerebral artery M1 trunk segment, *M2* middle cerebral artery M2 branch segments, *Mbif* middle cerebral artery bifurcation, *MCA* middle cerebral artery, *mRS* modified Rankin Scale, *Resi* visual estimate of the aICH volume reduction in the first CT after the evacuation, *sIA* saccular intracranial aneurysm

### Reconstruction of the 54 individual timelines since the EMS call

We collected from all available sources (EMS charts; CT, MRI, angiography; intensive care; interventions; hospital case reports) the defined time points to reconstruct the 54 individual clinical timelines. The timelines allowed to compare the individual time intervals from the 112 call to the aICH evacuation, also according to the weekday, the office hours and the duty hours. The patients’ timelines were also analyzed for possible re-bleeds until the sIA occlusion (Table 1, Figs. 2, 3). The verified re-bleeds were seen in two consecutive CT scans and the suspected re-bleeds were considered as worsening of the clinical condition (seizure, unconsciousness, dilated pupil).

### Individual serial CT/MRI scan panels for the 54 patients

The KUH digital image archive (PACS) is linked to the four Central Hospitals in Eastern Finland [2]. We were able to retrieve and review all CT scans, MRI scans and angiographies in the five digital archives of the 54 patients, using their personal identity codes. Figure 2 presents in the vertical columns the representative axial CT slices according to the increasing largest area of the aICH (calculated in PACS) on admission (Patients 1.–54.) and after the aICH evacuation, to evaluate the degree of aICH removal. The volumes of the 54 primary aICHs were calculated from the digital CT scans in the KUH PACS by (i) the ellipsoid volume formula π × (a × b × c) / 6 (*n*=48) or (ii) as the total aICH area in the slices × the slice thickness (*n*=6). For each patient, two axial CT slices representative of the aICH volume were selected: CT 1. before the clipping and CT 2. after the clipping. The residual aICH volume (Resi) in the first postoperative CT scan was visually graded (0 to 5) as follows: 0—no residual; 1—patchy small residual; 2—patchy residual; 3—small solid residual; 4—partial solid residual; 5—all solid left (Figs. 2, 4). The latest available slice (CT or MRI) was selected for the 38 survivors at about 12 months (not available in the surviving Patients 4. 15. 18. 19.).

### Literature review

PubMed was searched with the words (aneurysm* AND (intracerebral AND (hemorrhage OR hematoma)) AND (evacuation OR removal OR evacuated OR removed)) for English articles published between 2000 and August 2023. We excluded the single patient case reports, duplicate publications, systematic reviews, and meta-analyses. This gave 123 hits. The available articles were reviewed for the illustrated cases with a CT scan slice before and after the aICH evacuation, illustration the degree of aICH volume reduction in each case. The 12 approved articles are presented in Table 2. Additional articles with panels were not found by entering the aSAH patients’ individual brain CT/MRI panels from our three previous articles [2, 3, 72] and the current article into the Google Lens image recognition system (www.google.fi).

**Table 2:** Literature search for English articles from 2000 to August 2023 on patients with the microsurgical evacuation of an aneurysmal intracerebral hematoma (aICH). Single patient case reports were excluded. The available articles were reviewed for the illustrated cases with a CT scan before and after the aICH evacuation, presenting the degree of aICH volume reduction

Search sentence	Patients
Aneurysm* AND (intracerebral AND (hemorrhage OR hematoma)) AND (evacuation OR removal OR evacuated OR removed) Hits, n=123	Illustrative cases with a CT scan before and after the aICH evacuation n=21
Bohnstedt BN et al. (2013) [5]	1
Chung J et al. (2009) [11]	2
Fukuda H et al. (2015) [20]	1
Goren O et al. (2013) [28]	1
Horowitz ME et al. (2022) [33]	1
Jeong JH et al. (2007) [37]	2
Kazumata K et al. (2010) [38]	2
Niemann DB et al. (2003) [53]	2
Stapleton CJ et al. (2015) [69]	1
Turner RD et al. (2015) [76]	3
Wang MQ et al. (2018) [80]	3
Yang Y et al. (2022) [84]	2
The present cohort	54

*aICH* aneurysmal intracerebral hematoma, *CT* computed tomography

### Statistical methods

The categorical variables were expressed in proportions, and the χ2 test was used in comparisons. The continuous variables were expressed in medians, quartiles, and ranges, and the Mann-Whitney U test and the Spearman's *ρ test* were used in comparisons. The various time periods between time points were expressed in minutes, hours, days or months, and their distributions were presented with five times, as follows: (shortest – 25% quartile – median – 75% quartile – longest). Univariate analysis was used to analyze factors that associated to the aICH volume and the degree of aICH removal. *P* values < 0.05 were considered significant. We used the SPSS 27 statistical software (SPSS, Inc., Chicago, IL).

### Ethical aspects and pseudonymisation

The KUH Research Ethics Committee approved the study. KUH Neurosurgery IA Study Group had received a written informed consent from all patients in the database. The Ministry of Social Affairs and Health of Finland approved the data fusion from the national registries. The patients of the study cohort were not contacted during the study. In this article, we present *pseudonymized* data only on the 54 patients. Consequently, we excluded from the CT/MRI slice panel, tables, and texts the following data: name; gender; date, month and year of aSAH; clock times; time period lengths (except the time to death in Fig. 2); names of the referring hospitals [2]. The Kuopio IA Database does not contain face photos. The CT and MRI slices, 2 or 3 per patient (Fig. 2), do not allow the individual face recognition. The data presented does not yield *the correct attribution to an individual patient* (name; personal identity code; face photo or video) without the additional information strictly kept by us within the KUH Information System.

## Results

### Study population

In a total of 54 (65%) Mbif sIA patients with aICH (Patients 1.–54.), the hematoma was evacuated (reduced or seemingly removed) during the microsurgical clipping of the ruptured sIA (Table 1, Figs. 1, 2). There were 6 deaths until 14 days and a total of 12 deaths until 12 months. The remaining 29 (35%) Mbif aICH patients did not undergo an acute aICH evacuation in association with the sIA clipping (Fig. 1). The causes were very poor condition on admission (*n*=23), good condition with a small aICH (*n*=3), and undefined (*n*=3).

### Individual timelines and re-bleeds from the EMS call (112) to the aICH evacuation

The 54 individual times from the 112 call through the EMS transfer until the aICH evacuation at KUH distributed as follows: 1–4–**8**–18–43 hours (shortest – 25% quartile – **median** – 75% quartile – longest). In 8 patients the times exceeded 24 hours, in association with a small aICH (Patients 5. 6. 7. 8. 9. 33.), good condition (Patient 39.), or poor initial condition (Patient 46.). Of the 54 aICH evacuations, 27 (50%) were started in the office hours (Monday to Friday, 8–16). The time to the sIA clipping significantly shortened with the increasing aICH volume (*p* < 0,001) (Fig. 3). A total of 25 (46%) patients had a suspected (*n*=16) or CT-verified (*n*=9) re-bleed until the sIA occlusion: one per 11 hours of the total exposure time of 287 h. The aICH volume associated with the re-bleed (*n*=25; *p*=0,006) before the sIA occlusion, the presence of aIVH (*n*=17; *p*=0,017) and HH on admission (*p*=0,001) but not with the sIA diameter. Seven (28%) of the 25 patients with re-bleed vs. five (17%) of the 29 patients with no re-bleed deceased.

### Individual serial CT/MRI slice panel

Figure 2 presents the representative axial CT slices on admission (Patients 1.–54.) and after the aICH evacuation, (Table 1, Fig. 2). In the 42 survivors, the latest brain imaging to evaluate the final brain tissue outcome (not available in Patients 4. 15. 18. 19.) was CT in 24 patients (median 18 months) and MRI in 14 patients (median 19 months). In the 23 survivors, the time to the latest brain CT or MRI exceeded 12 months.

### Mbif sIA clipping and aICH removal

There were 30 right and 24 left ruptured Mbif sIAs, with a diameter distribution of 3-7-**9**-14-30 mm, one of them giant (Patient 42. 30 mm). In the preoperative angiography, the aneurysm neck seemed to involve one or both M2 branches in 26 (48%) patients, in association to an increased sIA diameter (7-11-**14**-16-30 mm vs. 3-5-**7**-8-12 mm; *p*<0,001). EVD was installed in 37 (69%) patients, 16 in the admission, 15 during the craniotomy and 6 later. During the 54 craniotomies, there were 16 (30%) re-bleeds, temporary M1 occlusion was used in 46 cases (median 5 minutes), and ICG angiography was performed in 46 cases.

Of the 54 aICHs, 39 (72%) were in the temporal lobe while 15 (28%) had crossed the Sylvian fissure into the frontal lobe. Virtually all aICHs were described as aspirable but the intrasylvian clot often remained (Fig. 2). The aICH volume reductions by visual grading are present in Figure 4. The primary aICH volume did not associate with the residual grade. In 47 patients (8. to 54.) the aICH appeared expansive, shifting the midline structures (Fig. 2). The median time to the start of the aICH evacuation was 16 hours for the HH 1-3 patients but only 5,5 hours for the HH 4–5 patients.

### Postoperative angiography and MCA branch occlusion

Postoperative angiography was available in 51 of the 54 patients. An M2 branch was found occluded in four patients (26. sIA 16mm; 30. 6mm; 36. sIA 15mm; 46. sIA 7mm), all, expect in Patient 30., the sIA neck was found embedded in the preoperative angiography. The occlusions were unexpected as the M2 branches were open in the ICG angiography or by micro-Doppler. Endovascular M2 thrombectomy was successful in one patient (26.) only. The M1 trunk was thrombosed in two patients (42. sIA 30mm; 43. 17mm) (Fig. 2) after proximal intraoperative rupture and difficulty in keeping the M2 branches open in spite of multiple clip adjustments.

### Brain expansion after the aICH removal and secondary DC

After the aICH removals, the reduction of brain expansion (reduced midline shift; increased ipsilateral ventricular volume) remained modest only, even in large aICHs. One reason was the development of perihematomal edema (PHE). The brain expansion seemed to reduce in seven patients only (alive: 12. 37. 40. 49. 53.; dead: 34. 46.). In 11 (20%) patients (alive: 8. 20. 25. 26. 30. 37. 41. 47.; dead: 36. 44. 52.), the aICH removal was followed by a secondary decompressive craniectomy (DC) at <1–1–**2**–3–4 days due to intractable ICP. Three of the four patients with an M2 occlusion had DC (Patients 26. 30. 36.).

### Clinical condition of the 42 surviving patients at 12 months

Of the 42 survivors (Fig. 2), 22 (52%) had a favorable condition (modified Rankin Scale, mRS, 0–2) at 12 months. Their characteristics distributed as follows: median Glasgow Coma Scale (GCS) 14 at EMS contact; 6 / 22 re-bleeds before sIA occlusion; HH scale on admission: 1 HH1 – 2 HH2 – 11 HH3 – 6 HH4 – 2 HH5; median aICH volume 33 cm^3^; 3 / 22 with aIVH clot; 1 / 22 with tracheostomy (patient 24.); median neuro ICU time 8 days; 2 / 22 with DC (and later cranioplasty; Patients 8. 20.); 5 / 22 with shunt (Patients 10. 14. 17. 21. 28.). Their CT/MRI scans at about 12 months (brain tissue outcome) displayed modest loss of brain tissue at the sites of the aICHs. On the other hand, four (10%) survivors (Patients 38. 40. 49. 53.) remained in hospice care (mRS 5), in association with larger aICH volumes (median 74 cm^3^).

## Discussion

Our aim was to illustrate the individual clinical courses of 54 consecutive aICH patients who acutely underwent a microsurgical clipping of the ruptured middle cerebral artery bifurcation (Mbif) sIA together with the evacuation of an adjacent aICH. Here we visualize in real life the shapes and volumes of the 54 aICHs, the impact re-bleeds, the true degrees of aICH evacuations, the development of brain edema, PHE and DCI, and finally, the brain tissue injuries around the aICH cavity at a median of 18 months (*final brain tissue outcome)* (Fig. 2) [45, 48]. We find that our serial brain CT/MRI panel, with the individual patient numbers, presents easily more visual and comparative information on the aICH course than any rational amount of words, figures and graphs would convey [2, 3, 72]. This approach would support Personalized Neuroacutology in general, also in a published form.

### Construction and pseudonymization of the individual serial brain CT or MRI slice panels

We constructed a serial brain CT/MRI slice panel for the 54 patients [2], each slice in an attempt to illustrate the largest aICH volume, PHE, DCI, and the final brain tissue outcome. There was unfortunate shortage of MRI scans, none obtained close to the admission. In this article, we present pseudonymized data only on the 54 Mbif aICH patients. The representative CT or MRI slices do not allow the individual face recognition, e.g., via the facial photos or videos provided in the social media [73]. For the General Data Protection Regulation (GDPR) compliant pseudonymization [18] we excluded from the CT/MRI slice panel, tables, and texts the following data: name; gender; date, month, and year of aSAH; clock times; and time period lengths (except the time to death in Fig. 2).

### PubMed and Google Lens search for serial brain imaging panels illustrating aICH evacuations

It is evident that acute aICHs are sidelined in neurovascular literature in many ways. The frequently used Fisher scale (>700 articles since 1988 in PubMed), focused on blood in the cisternal, intrasylvian and ventricular CSF spaces, does not specify (e.g., location, size, shape, extensions) aICH or aIVH ‘clots’ at all [49, 107]. PHE with secondary brain injury around aICH receives little attention: PubMed gave 406 hits on PHE but only two when adding SAH [2, 39]. A recent guideline on the neurointensive care in aSAH does not discuss aICH or PHE [74]. We found only 12 articles that illustrated the aICH evacuation in a total of 21 patients (Table 2). Google Lens suggested several brain CT or MRI panels from other brain diseases, articles, and sources, but none of them did illustrate the degrees of aICH evacuations. Importantly, there was no linkage to the identity of any of our 54 patients. In contrast, Case Reports and Illustrative Cases with a lot of focused data on one or few patients might become vulnerable to the Artificial Intelligence (AI) decryption, e.g., via social media, local or national media, personal news, CVs, obituaries, et al.

### aICHs at the MCA bifurcation

The MCA bifurcation is a frequent site for the ruptured sIAs [15, 17, 52]: 24% in our basic series of 613 aSAH patients between 2010 and 2019 (Fig. 1). The Mbif sIAs are within the Sylvian fissure, between the frontal and the temporal lobe, and this setting may predispose to aICHs. Of the 146 ruptured Mbif sIAs, as many as 83 (57%) had caused an aICH (Fig. 1): 72% remained in the temporal lobe while 28% extended into the frontal lobe (Fig. 2).

### Individual timelines from the EMS call (112) until the aICH evacuation

The individual timelines of the aSAH patients, with or without aICH, start from the ictus and the EMS call, not from the tertiary hospital door [2, 3, 42, 67, 68]. The KUH catchment area and the ambulance transfer distances from the four local hospitals until KUH are presented in our previous article [2]. Figure 3, possibly for the first time in aSAH literature, plots the present 54 aICH volumes against the hours from the EMS call until the start of the aICH evacuation. The plot shows how larger volumes associated with (i) the re-bleeds and (ii) the deaths [2]. In a Norwegian cohort of 486 aSAH patients, with the EMS logistics of the defined catchment area, 9.7% had re-bleeds between the ictus and the aneurysm repair, and the frequency of re-bleeds increased from H&H grades 1 to 5 [67, 68]. In an Italian study of 443 poor grade (World Federation of Neurosurgical Societies, WFNS, IV-V) aSAH patients, re-bleeds occurred in 17.6% and were an independent risk of in-hospital mortality [56].

Concerning re-bleeds, there were 9 CT verified re-bleeds and 16 clinically suspected re-bleeds (worsening of the clinical condition, seizure, unconsciousness, dilated pupil). The question arises whether all the 16 clinical re-bleeds were true re-bleeds (Sorteberg et al. 2021) or in some cases the brain’s acute responses to aSAH, very high ICP, acute hydrocephalus, decreased perfusion, etc. [68]. Our data is not conclusive. The median aICH volumes were 81 cm^3^ in 9 CT-verified re-bleeds vs. 68 cm^3^ in 16 clinical re-bleeds vs. 44 cm^3^ in 29 cases with no re-bleeds. Dubiously, 81 cm^3^ vs. 68 cm^3^ would speak against clinical re-bleeds while 68 cm^3^ vs. 44 cm^3^ would support them.

Figure 3 also shows that larger aICH volumes associated with shorter times until the aICH evacuations: this reflects the swift and mutually honed EMS logistics in the KUH catchment area when faced with a sinister ICH in the first CT and teleconsultation [2]. Time is brain [34]. Accordingly, 27 (50%) aICH evacuations were started in the duty hours or in the weekends [24].

In spontaneous supratentorial ICHs (sICHs), for comparison, the goal time since the 112 call is 8 h in the Dutch ICH Surgery Trial (NCT03608423) [66], and CT-neuronavigated minimally invasive neuroendoscopic evacuation is recommended [29]. Further studies would find out which sICH patients benefit from early surgery.

### Degrees of aICH evacuations

It is difficult to define which degree of evacuation would be optimal in various sites and sizes of sIAs and aICHs [84]. Minimal residual in the control CT would seem natural but illustrated cohorts are virtually lacking. In a Chinese single-center cohort of 358 aICHs evacuated along the clipping, 265 (74%) had a residual hematoma in the postoperative CT [84]. The authors notified that excessive pursuit of aICH evacuation would lead to neurovascular injuries [84]. In our cohort, the degrees of aICH evacuations did not seem optimal in that there were unexpectedly large residuals (Fig. 2). There were several caveats: (i) 27 evacuations in duty hours; (ii) lack of acute CT-based neuronavigation [29]; (iii) intraoperative brain edema and re-bleeds; (iv) hesitancy of clot removal in the Sylvian fissure; (v) lack of intraoperative CT [30]. Five microvascular surgeons of the KUH Neurovascular Group operated on 49 of the 54 patients.

### Challenges of ruptured Mbif sIA clipping

The Mbif sIA neck seemed to involve one or both M2 branches in 26 (48%) patients, along with an increased sIA diameter (*p*<0,001). This necessitated repeated clip positionings under temporary M1 occlusion to ascertain open M2 branches, often under challenging intraoperative conditions [16, 51, 78]. In postoperative angiography, an M2 branch was occluded in four patients, unexpectedly as the M2 branches were open in the ICG angiography or by micro-Doppler. Endovascular M2 thrombectomy was successful in one patient (26.) only [35].

### Intraoperative adverse events (AEs)

In a Swedish prospective nationwide aSAH cohort (2014–2018), 322 (31%) patients underwent microsurgical treatment in a median of 18 hours after the admission: 83% with Fisher grade 4; 18% with an aICH evacuation; 83% with a wide IA neck [4, 64]. Intraoperative AEs occurred in 79 (25%) patients: re-repture (13%); temporary parent artery occlusion > 5 min (8%); adjacent vessel injury with cerebral infarction (8%). Microsurgery was preferred when EVT seemed too difficult or risky, or aICH needed evacuation. The authors suggest that an alternative method of the IA occlusion, such as EVT, should be considered with high Fisher grade and/or brain edema. Considering ruptured MCA bifurcation sIAs, with or without aICHs, it remains to be seen whether acute EVT proves generally successful and reliable [14]. To learn, maintain and advance microneurovascular proficiency, only sub-specialized neurosurgeons with structured training [77], in-house training facilities [23, 26, 78], intraoperative monitoring [16, 82], AI supported conduct of care [48] and sufficient case-volumes [44] should operate on aSAH patients, without or with aICHs, regardless of duty hours or days of week [2, 24].

### A hybrid operation room for ICHs and IVHs of various etiologies

In theory, a comprehensive (i) microsurgical, (ii) endovascular and (iii) cone beam CT (CBCT) facility would offer a fast track for spontaneous, aneurysmal, AVM-derived and DAVF-derived ICHs and IVHs, with CT conforming (a) the degree of hematoma removal and disclosing (b) possible re-bleeds. Robotics in neuroendovascular surgery would allow very high accuracy and precise movements difficult to achieve manually [7]. A portable low-field MRI scanner might provide repeated brain scanning for PHE and brain ischemia [1]. Hybrid ORs, quite frequent in spinal surgery, are also used to some extent in neurovascular surgery [23]. That would require significant case flows with swift logistics and experienced personnel: i.e., most neurovascular interventions to be performed there [50]. An important concern is radiation exposure [23].

### Perihematomal edema (PHE) and brain expansion after the aICH evacuation

PHE is a well-recognized and dangerous cause of secondary brain injury in spontaneous ICH (sICH) [12, 33, 66]. PubMed gave 406 hits on PHE but only two when adding SAH [2, 39]. In our cohort, 11 (20%) of the acute aICH evacuations were followed by a secondary decompressive craniectomy in a few days [2, 6]. The overall mechanisms of PHE are complex and not well-defined. So far, the available option would be—within reason—prompt (i) evacuation of blood from the aICH cavities and (ii) rinsing with catheters from cisterns [19, 22, 83] and ventricles [36].

### Strengths and limitations of the study

The strengths derive from the tax-paid Finnish health care system and the automatic archival of clinical data, using the Finnish identity codes, in the national registries. Finland is divided into exclusive catchment areas between the five university hospitals which results in cohorts that are minimally selected and biased. The Kuopio Database contains all aSAH patients admitted from Eastern Finland and allows to reconstruct their individual clinical timeline and serial brain imaging panels, including data from other hospitals and national registries.

There are also limitations. Our study is retrospective while the database was prospective in the study period. No intraoperative videos were available to evaluate the aICH evacuation. There was a shortage of MRI scans, preventing serial PHE volumetry from T2 and FLAIR scans [9, 85]. There was no regular and mutually accepted imaging follow-up schedule in the five hospital districts of Eastern Finland during the study period. In four of the 42 survivors there was no available follow-up CT or MRI scan.

### Suggested further development

1. In clinical aSAH articles, any findings associated with (i) the final patient outcome [54] and (ii) the final brain tissue outcome could be illustrated with pseudonymized patients’ individual timeline panels and serial brain imaging panels [2, 3, 72]. This approach would support Personalized Acute Neurocare.
2. It would be straightforward—as soon as possible after the emergency call—to relieve the brain from (i) the aICH and aIVH clots by evacuation and from (ii) the blood containing CSF by ventricular, cisternal and lumbar drainage [19, 22, 36, 83]. In theory, a hybrid microvascular and endovascular operating room would allow minimal delay, a preferred IA occlusion method, and endoscopic or open hematoma evacuation.
3. The mechanisms of PHE provoked by intracerebral hemorrhages are complex and not well-defined. Serial brain tissue monitoring with a portable low-field MRI scanner could illustrate the timelines and dynamics of PHE, not fittingly shown by serial CT [85]. Biologically targeted novel drug therapies to inhibit neuroinflammation and PHE from the very start are warranted [2]. Continuous infusion of novel drugs into main cerebral arteries, cisterns, and ventricles is conceivable [36].

## Clinical conclusions

1. Regarding the impact of aICH evacuations, serial brain CT/MRI panels, with individual patient numbers, present more visual and comparative real-life information since the emergency call than words, figures, and graphs.
2. Clinical or verified re-bleeds associated with larger aICH volumes and worse outcomes. Swift logistics in the served population until the sIA occlusion and the aICH evacuation required, also in duty hours and weekends. Intraoperative CT is needed to illustrate the true degree of aICH evacuation.
3. Perihematomal edema (PHE) may evoke uncontrollable ICP in spite of the acute aICH volume reduction. Novel biologically targeted approaches to inhibit PHE with any ICHs from the very start are warranted.

## Data Availability

All research consents, data, material, and coding are available for corresponding author.

## Abbreviations

AE: adverse event
AI: artificial intelligence
aICH: aneurysmal intracerebral hematoma
aIVH: aneurysmal intraventricular hematoma
aSAH: aneurysmal subarachnoid hemorrhage
AVM: arteriovenous malformation
CBCT: cone beam computed tomography
cm^3^: cubic centimeter
CSF: cerebrospinal fluid
CT: computed tomography
CTA: computed tomography angiography
D: deceased
DAVF: dural arteriovenous fistula
DC: decompressive craniectomy
DCI: delayed cerebral ischemia
DSA: digital subtraction angiography
EMS: emergency medical services
EVD: extraventricular drainage
EVT: endovascular treatment
GCS: Glasgow Coma Scale
GDPR: General Data Protection Regulation
GPS: Global Positioning System
HEMS: helicopter emergency medical services
HH: Hunt and Hess scale
IA: intracranial aneurysm
ICG: indocyanine green angioprahy
ICHE: aneurysmal intracerebral hematoma evacuation
ICP: intracranial pressure
ICU: intensive care unit
KUH: Kuopio University Hospital
M1: middle cerebral artery M1 trunk segment
M2: middle cerebral artery M2 branch segments
Mbif: middle cerebral artery bifurcation
MCA: middle cerebral artery
Mdist: middle cerebral artery peripheral segments
MRI: magnetic resonance imaging
mRS: modified Rankin Scale
PACS: picture archiving and communication system
PHE: perihematomal edema
rb: re-bleed before the sIA occlusion
Resi: visual estimate of the aICH volume reduction in the first CT after the evacuation
SAH: subarachnoid hemorrhage
sIA: saccular intracranial aneurysm
sICH: spontaneous intracerebral hematoma
UMC: University Medical Center
WFNS: World Federation of Neurosurgical Societies.
